# Association between snack intake behaviors of children and neighboring women: A population-based cross-sectional analysis with spatial regionalization

**DOI:** 10.1016/j.ssmph.2024.101720

**Published:** 2024-10-13

**Authors:** Emiko Yamamoto, Daisuke Takagi, Hideki Hashimoto

**Affiliations:** Department of Health and Social Behavior, School of Public Health, The University of Tokyo, 7-3-1 Hongo, Bunkyo-ku, Tokyo, 113-0033, Japan

**Keywords:** Snack intake, Neighborhood environment, Spatial regionalization, Dietary behavior, Elementary schoolchildren, Social learning

## Abstract

**Background:**

Accumulated evidence indicates that neighborhood environments affect children's health behaviors. However, measuring neighborhood environments remains challenging because there exist strengths and weaknesses both in objective and perceived environment measures. Drawing on a recent conceptual model of how environment, perception, and behavior interact, we hypothesized that neighbors' behavioral similarities indicate the combined influence of physical and social environmental opportunities on specific behaviors. We then examined how these similarities (i.e. the behavioral tendencies of children's adult neighbors) relate to children's obesogenic dietary behaviors.

**Methods:**

We used data for 2275 women and 821 elementary schoolchildren from a 2012–2013 population-based survey in greater Tokyo, Japan. Snack intake was defined as the total consumption of various types of snacks, estimated using a validated self-administered diet history questionnaire. Spatial regionalization, a type of spatial clustering, was used to empirically identify segments that could effectively differentiate regional variation in women's snack intake behaviors. We conducted multiple regression analysis to assess the cross-sectional association between children's snack intake and the mean snack intake of neighborhood women, adjusting for mother's intake.

**Results:**

A 1-g increase in the mean snack intake of neighborhood women was associated with a 0.23-g (95% confidence interval: 0.00–0.45) increase in children's intake, while a 1-g increase in mother's intake was associated with a 0.34-g (95% confidence interval: 0.26–0.41) increase in children's intake.

**Discussion:**

The results suggest that the out-of-home physical and social neighborhood environments may have non-ignorable associations with children's dietary behaviors by offering behavioral opportunities in addition to maternal influence.

## Highlights

• Detecting environmental effects on health behaviors is methodologically challenging.
• We proposed a concept of behavioral opportunities of neighborhood environments.
• We operationalized neighbors' behavioral similarities as an index of opportunities.
• Snack intake in neighborhood adults was related to children's snack intake.
• The behavioral opportunities of neighborhood environments warrant more research.

## 1 Introduction

Neighborhood environment is considered a strong influencer on health behaviors, including dietary behaviors (Arcaya et al., 2016; Diez Roux & Mair, 2010; Glanz et al., 2015; Macintyre et al., 2002; Oakes et al., 2015). Research findings on physical activity have recently been translated into policy recommendations to create supportive environments for health promotion (Dixon et al., 2021; World Health Organization Regional Office for Europe, 1997; Pontin et al., 2022; Smith et al., 2017; Zhong et al., 2022). However, the available evidence for the dietary health-promoting effects of built environments is inconsistent (Atanasova et al., 2022; Bivoltsis et al., 2018; Cobb et al., 2015; Daniels et al., 2021; Feng et al., 2010; Lam et al., 2021; Travert et al., 2019; Wilkins et al., 2019; Xin et al., 2021).

Previous reviews have indicated that one reason for these inconsistent findings is methodological heterogeneity in assessing environmental characteristics across studies (Engler-Stringer et al., 2014; Feng et al., 2010; Schule & Bolte, 2015; Wilkins et al., 2019).

One methodological issue is the use of different measurement constructs of environmental exposure (Feng et al., 2010). Previous studies have exclusively used the physical structural aspects of the environment (e.g., existence of built environment) as a surrogate measure of environmental exposure, by assuming that geographical closeness to built structures reflects resource accessibility that facilitates behavioral implementation (Larson et al., 2009). However, although the existence of the built environment per se is objectively identifiable, it may not be equally influential on different individuals’ behavioral intentions and felt efficacy in a potentially affected geographical region (Travert et al., 2019). An alternative approach is to measure the perceived availability of the built environment to capture the individual-level effects of the environment. However, this measure is affected by individual-level (non-environmental) confounders such as the demographic, socioeconomic, and functional characteristics of individuals (Diez Roux & Mair, 2010; Travert et al., 2019).

In this study, we applied the above argument to the case of snack intake behavior among elementary schoolchildren. The snack intake of children is a major target for behavioral interventions to prevent childhood obesity (Larson & Story, 2013; World Health Organization, 2017). Children's obesogenic dietary behaviors are affected by various environmental factors, such as food availability at school and marketing of unhealthy foods (Alcaire et al., 2021; Briefel et al., 2009; Chacon et al., 2013), as well as by individual characteristics of children and their families (Blaine et al., 2017). These environmental factors have been targeted in recent community- and place-based interventions for preventing childhood obesity without stigmatizing individual children (World Health Organization, 2012, 2017).

We used a theoretical model proposed by Travert et al. (2019) to conceptualize the effect of the environment on a targeted behavior. This model clarifies the interactions between built environments, perception, and behaviors (Travert et al., 2019). It posits that both the external physical environment and the subjectively perceived environment interactively shape behavioral opportunities, which either facilitate or hinder behavioral motivation. Drawing on this model, we hypothesized that neighbors’ behavioral similarities are an indicator of the behavioral opportunities that neighborhood environments, both physical and socially perceived, can offer. Our rationale was that the behavioral propensity of surrounding residents may embody indicative opportunities for targeted behaviors prevalent within the community residents, reflecting the collective effect of both external and internal environmental influences. Considering the interconnectedness of physical and social environments (Suglia et al., 2016; Travert et al., 2019), such behavioral opportunities may reflect the integrated effects of shared physical and social environments in the neighborhood.

## 2 Methods

### 2.1 Data

This study was based on the Japanese Study on Stratification, Health, Income, and Neighborhood (J-SHINE) project, which is an ongoing longitudinal panel study of households. A detailed study profile has been described elsewhere (Takada et al., 2014). The Wave 1 survey was conducted in four urban and suburban municipalities in the greater Tokyo metropolitan area, Japan, in 2010. Community-dwelling adults aged 25–50 years were selected from the residential registry using probability sampling (response rate: 51.8%). An additional survey of the participants’ spouses/partners and children was conducted in 2011 (response rate: 61.9% for spouses/partners and 67.7% for children). The Wave 2 survey was conducted in 2012 for the original Wave 1 participants and in 2013 for the spouses/partners and children. The present study mainly used cross-sectional data obtained in the Wave 2 survey, which involved a dietary survey completed by 2825 original participants, 1706 spouses/partners, and 1503 children.

For this study, we selected women (n = 2363) and elementary schoolchildren (aged 6–12 years; n = 868) who answered a food frequency questionnaire in the Wave 2 survey. Of these, we excluded participants who reported extremely low or high energy intake (women: n = 64; children: n = 39), using the proposed outlier criteria in a previous study (Sasaki et al., 2003). We also excluded individuals who were outliers for reported snack intake (more than ±3 standard deviations from the mean) (women: n = 24; children: n = 8). Finally, data for 2275 women and 821 elementary schoolchildren were used for the analysis.

The study protocol was approved by the ethics committee of the Ethics Committee of the Graduate School of Medicine of The University of Tokyo. Written informed consent was obtained from all participants.

### 2.2 Measurements

#### 2.2.1 Target variable: snack intake

The dietary intake of the adult participants was estimated using the brief-type self-administered diet history questionnaire for Japanese participants (BDHQ) (Kobayashi et al., 2011, 2012; Okubo et al., 2008). For child participants, the BDHQ 10y/15y was self-administered or administered with parental support (Okuda et al., 2009).

The BDHQ 10y/15y categorizes confectionery and snacks into six groups: 1) Western confectionery (e.g., cookies and biscuits); 2) Japanese confectionery; 3) other confectionery (e.g., rice crackers); 4) ice cream; 5) snacks; and 6) chocolates. The BDHQ categorizes these foods into four groups: 1) Western confectionery (e.g., cookies and biscuits); 2) Japanese confectionery; 3) other confectionery (e.g., rice crackers); 4) ice cream. We defined snack intake as the sum of consumption of all six BDHQ 10y/15y categories and all four BDHQ categories. The relative validity of the total snack intake was indicated by the correlation of this variable with weighed dietary records in a previous study (Kobayashi et al., 2011).

For analysis, all snack intake was energy-adjusted using the density method, and estimated as a value (g) per 1000 kcal of daily total energy intake (Willett et al., 1997).

#### 2.2.2 Environmental variables

##### 2.2.2.1 Neighborhood behavioral tendencies as a surrogate index of behavioral opportunities

According to the social cognitive theory (Bandura, 1977), an individual's physical and social environments exert effects on their health behaviors through physical conditioning of their behavioral tendencies (i.e., availability of enabling resources and built conditions), normative beliefs (i.e., social acceptability and attitudes toward a behavior, as shaped by the perceived prevalence of the particular behavior in the community), and observational learning (i.e., learning a particular behavior by observing the behaviors of others, such as community members or influential persons in the individual's environment). These environmental factors may affect personal cognitive factors (e.g., self-efficacy, outcome expectations, and knowledge), ultimately determining the personal motivation to engage in a particular health behavior (Glanz et al., 2015).

In learning health-related behaviors, children are particularly sensitive to their environment (Brooks-Gunn et al., 1993; Diez Roux, 2001; Diez Roux & Mair, 2010; Minh et al., 2017). Previous studies have indicated that maternal dietary behaviors strongly affect the development of children's obesogenic dietary behaviors (Johnson et al., 2011; Vaughn et al., 2018). We further reasoned that children also observe the behaviors of neighborhood adults. Children learn social norms and how they should behave from these perceived behaviors.

The neighborhood physical and social environment influences both children and neighborhood adults, but each individual's behavior is modified by various factors, including behavioral opportunities and motivations. As discussed in Section 1, we hypothesized that neighbors' behavioral similarities can indicate the combined influence of physical and social environmental opportunities on a targeted behavior in the community. If observational learning in children also occurs through observing neighborhood adults, there may be a relationship between the mean dietary behavior of neighborhood adults and the dietary behavior of children, through the combined influence of shared physical and social environmental opportunities, as well as through observational learning. Therefore, we hypothesized that the dietary behavior patterns of neighborhood adults may be positively associated with children's dietary behaviors.

##### 2.2.2.2 Rationale for selection of neighborhood women's behaviors

We selected women rather than all adults in the neighborhood for two reasons. First, evidence suggests that women are more sensitive to their neighborhood environments than men (Stafford et al., 2005). Second, within the Japanese cultural context, men are usually assumed to be the breadwinners and women's labor participation is relatively limited to part-time employment because of childcare duties (Umeda et al., 2015); therefore, women spend more time in their neighborhood than men. Indeed, a national social survey reported a lower proportion of full-time workers (National Institute of Population and Social Security Research, 2020) and a shorter weekly commute time (Statistics Bureau of Japan, 2017) in women than in men. Therefore, we assumed that neighborhood women were more frequently observed by children during their daily lives (Ministry of Health, Labour and Welfare of Japan, 2019) and were more representative of the net effect of the neighborhood food environment than men.

##### 2.2.2.3 Identification of spatial boundaries of an environment

The operational identification of environmental boundaries is an important methodological issue in research on neighborhood environments (Feng et al., 2010; Macintyre & Ellaway, 2003). Previous studies have tended to use predetermined administrative boundaries, such as census tracts, or relatively small areas created by geographic buffers based on individuals. However, in studies examining the association between neighborhood characteristics and diet, there are often discrepancies between how researchers and community residents define neighborhood boundaries (Larson et al., 2009). Recent arguments in spatial epidemiology have criticized the arbitrary choice of boundary segments as a source of statistical bias called the modifiable areal unit problem (Flowerdew et al., 2008; Mobley et al., 2008).

Some previous studies have proposed that regional boundaries can be empirically defined by referring to the commonality of environmental/social exposure status in the community (Takagi et al., 2012; Wei et al., 2016). Regionalization techniques and other empirical approaches have been posited as alternative methods of defining areal segments that effectively explain the regional differences in factors related to target outcomes (Guo & Wang, 2011; Santos et al., 2010; Schaefer-McDaniel et al., 2010; Wang, 2020; Wei et al., 2016). Regionalization is a special type of spatial clustering technique based on geographic connectivity, in addition to data coherence within a cluster. Each spatial cluster is delineated by aggregating smaller geographical areas into a larger region to maximize both the homogeneity within the region and the heterogeneity between regions in terms of a designated characteristic (Guo & Wang, 2011).

These approaches may be useful in studies focusing on people's health behaviors, particularly behaviors that may cause regional differences in a small areal unit, such as a neighborhood (Macintyre & Ellaway, 2003). The use of empirically defined boundaries for a targeted behavior may facilitate a more precise analysis of neighborhood environmental effects (Caughy et al., 2013). In addition, a recent study demonstrated the possibility that some spatial clustering can assist in the detection of regional variation in dietary patterns (Dekker et al., 2017). Therefore, in this study, we used a regionalization method and aggregated census tracts with behavioral similarity to identify new larger areas in which people express similar behavioral patterns, and defined these areas as empirical neighborhoods.

Specifically, we used the Spatial K'luster Analysis by Tree Edge Removal (SKATER) method (AssunÇão et al., 2006) because recent studies comparing regionalization methods (AssunÇão et al., 2006; Aydin et al., 2021; Guo, 2008) have shown that it is computationally cost-efficient and can reduce the sensitivity of the clustering procedure. Previous studies have confirmed that the SKATER algorithm can produce meaningful regions to explain regional patterns of unequal distribution of socially determined health outcomes (Santos et al., 2010; Wei et al., 2016).

##### 2.2.2.4 Procedure for creating environmental variables

We used regional mean women's snack intake as an environmental variable. We obtained geographical information data for city blocks, which were the unit of sampling in the J-SHINE survey. Using the SKATER method, we aggregated spatially adjacent city blocks into a larger agglomerate according to the mean women's snack intake of each city block. We defined these agglomerates as a new neighborhood unit for analysis. The technical details of the generation of new regional segments are provided in Appendix 1. To determine whether the new segments produced homogeneous within-cluster variance and heterogeneous between-cluster variance, we conducted an analysis of variance of women's snack intake. Finally, for each child, we calculated the mean snack intake of women, except the child's mother, in the obtained regional segment to which the child belonged as the environmental variable.

#### 2.2.3 Covariates

The covariates related to children's snack intake behavior included the child's sex, age, existence of siblings below elementary school age, mother's educational attainment, and mother's employment status, based on a previous study (van Ansem et al., 2014). Mother's educational attainment was categorized into four groups: 1) low (completed high school or less); 2) medium (vocational or junior college); 3) high (4-year college degree or more); or 4) missing. Mother's employment status was categorized into three groups: 1) full-time job or self-employed; 2) part-time job or other job; or 3) housewife or unemployed.

Dummy variables representing the municipality cities were included in the analysis to account for municipality-specific fixed effects, such as those of municipal government policy on dietary education for children at school, and other regionally specific factors.

### 2.3 Statistical analysis

We conducted a multiple regression analysis to assess the association between children's snack intake and the environmental variable. The inclusion criteria for the children were as follows: 1) no energy outliers or snack intake outliers for either the child or the child's mother; and 2) presence of more than three women in the regional segment to which the child belonged (e.g., the child's mother and more than two other women). Other variables were added as follows: environmental variable for Model 1; individual characteristics for Model 2; and city dummy variables for Model 3. Although multilevel analysis is often used to assess the effects of neighborhood variables on individual outcomes, we did not use this type of analysis because our environmental variable was tailored uniquely to each child by excluding their mother's snack intake.

Spatial analyses were performed using ArcGIS Pro (Esri, Redlands, CA, USA). Regression analyses were performed using Stata version 14 (StataCorp, College Station, TX, USA). Values of p < 0.05 were considered to indicate statistical significance.

## 3 Results

**Fig. 1:**
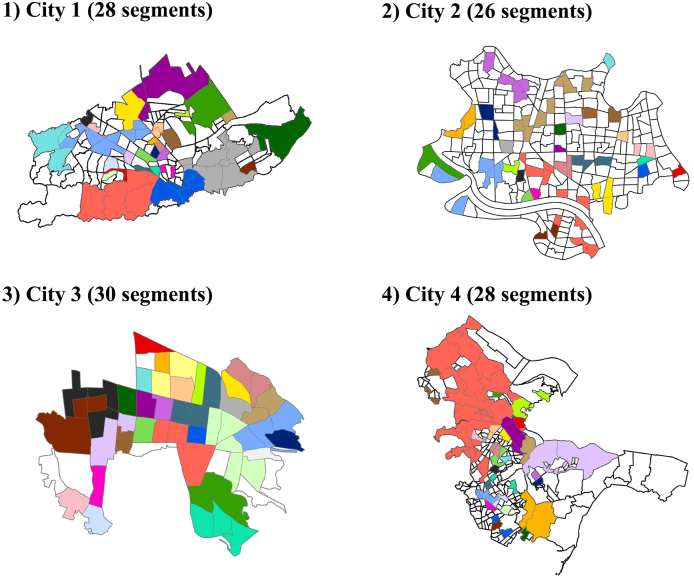
Obtained regional segments based on areal characteristics and spatial proximity of all cities. Areal characteristics were defined by the mean women's snack intake similarity. Each color indicates a different segment. As we had both unsampled and sampled blocks for some municipalities, if a block had no contiguous neighborhood blocks, we treated the nearest block using the center distance as a contiguous block. (For interpretation of the references to color in this figure legend, the reader is referred to the Web version of this article.)

The background characteristics of the neighborhood women (including the mothers of the analyzed children) whose data were used to generate the spatial segments are shown in Table A.1. Of the 60 city blocks originally sampled from each municipality in the J-SHINE survey, 53 city blocks from city 1, 60 from city 2, 53 from city 3, and 55 from city 4 were used in the following analysis. The background characteristics of the regional segments obtained using the SKATER method are shown in Table 1. We prepared 112 segmented spatial areal clusters (28 segments in city 1, 26 segments in city 2, 30 segments in city 3, and 28 segments in city 4; Fig. 1). The number of women in the segments ranged from 2 to 206, with a mean of 20.3. The intraclass correlation coefficient (ICC) for women's snack intake by the obtained regional segments (ICC = 0.057; 95% confidence interval [CI]: 0.025–0.088) was larger than those for the predetermined administrative boundaries of the city blocks (ICC = 0.016; 95% CI: 0.000–0.038) and municipality cities (ICC = 0.001; 95% CI: 0.000–0.005).

**Table 1:** Background characteristics of regional segments obtained using the SKATER method.

1) Background characteristics of women whose data were used to obtain the regional segments									
	Number of regional segments			Number of participants in obtained regional segments				Segment mean snack intake (g/1000 kcal)	
	Predetermined city blocks	City blocks selected in J-SHINE	Obtained regional segments	Mean	SD	Min	Max	Min	Max
City 1	160	53	28	22.9	29.2	7	113	22.9	62.6
City 2	269	60	26	17.0	18.7	4	97	21.2	61.1
City 3	62	53	30	16.9	14.3	2	60	19.6	58.9
City 4	287	55	28	24.4	37.2	7	206	26.3	66.4
Total	778	221	112	20.3	26.2	2	206	19.6	66.4

2) Variance in women's snack intake between administrative city blocks and obtained regional segments					
n = 2275	F-value	p-value	ICC	95% CI	
Between predetermined administrative city blocks	1.15	0.063	0.016	0.000	0.038
Between obtained regional segments	2.20	0.000	0.057	0.025	0.088
Between four cities	1.54	0.202	0.001	0.000	0.005

SD, standard deviation; ICC, intraclass correlation coefficient; J-SHINE, Japanese Study on Stratification, Health, Income, and Neighborhood; CI, confidence interval; SKATER, Spatial K'luster Analysis by Tree Edge Removal.

The background characteristics of the children (n = 821) are shown in Table 2. Data for 419 boys and 402 girls were analyzed. The mean age of the children was 9.3 years. The number of children in each age category was similar. The mean snack intake was 41.6±23.7 g/day. Children's snack intakes did not differ by sex, age, existence of siblings below elementary school level, mother's educational attainment, or mother's employment status, but did differ between municipality cities (F(3, 817) = 6.37, p < 0.001). The background characteristics of the children in the obtained regional segments are shown in Table 3. The number of children in the obtained regional segments ranged from 1 to 97, with a mean of 7.7. The minimum and maximum of the mean snack intakes of the children by the obtained regional segments were 11.1 and 85.4 g/1000 kcal respectively.

**Table 2:** Background characteristics of children and descriptive statistics for snack intake by each characteristic.

	Number	%	Snack intake (g/1000 kcal)		
			Mean	SD	p-value^a^
Total	821	100	41.6	23.7	
Sex					
Boys	419	51.0	40.4	23.8	0.154
Girls	402	49.0	42.8	23.6	
Age (years)					
6	23	2.8	47.8	18.1	0.824
7	145	17.7	40.6	23.7	
8	130	15.8	41.8	24.5	
9	142	17.3	40.1	23.9	
10	139	16.9	43.0	25.3	
11	137	16.7	41.2	22.2	
12	105	12.8	41.8	24.0	
Existence of siblings below elementary school age					
Yes	561	68.3	42.6	24.4	0.063
No	260	31.7	39.3	22.2	
Mother's educational attainment					
Low	232	28.3	43.5	23.9	0.151
Medium	344	41.9	41.3	24.1	
High	203	24.7	39.0	22.1	
Missing	42	5.1	45.8	26.9	
Mother's employment status					
Full-time job or self-employed	130	15.8	39.2	22.7	0.100
Part-time job or other job	347	42.3	43.3	24.2	
Housewife or unemployed	300	36.5	39.8	23.8	
Municipality					
City 1	213	25.9	41.8	23.3	<0.001
City 2	173	21.1	48.1	25.9	
City 3	167	20.3	39.2	23.0	
City 4	268	32.6	38.7	22.4	

SD, standard deviation.

a The p-values were calculated using analysis of variance.

**Table 3:** Background characteristics of children in the regional segments.

	Number of segments containing children	Number of children				Segment mean snack intake (g/1000 kcal)	
		Mean	SD	Min	Max	Min	Max
City 1	28	7.6	9.0	1	32	11.1	85.4
City 2	26	6.7	6.3	1	31	26.8	79.0
City 3	25	6.7	5.8	1	21	12.3	55.9
City 4	28	9.6	18.1	1	97	13.5	83.4
Total	107	7.7	11.1	1	97	11.1	85.4

SD, standard deviation.

Table 4 shows the results of the multiple regression analysis. The model that included only the environmental variable (Model 1) showed that the environmental variable was positively associated with children's snack intake (regression coefficient = 0.41; 95% CI: 0.19–0.64; i.e., a 1-g increase in mean snack intake of neighborhood women corresponded with a 0.41-g increase in children's intake). Model 2, which was adjusted for children's individual characteristics, indicated that a 1-g increase in neighborhood women's intake was associated with a 0.23-g increase in children's intake (95% CI: 0.00–0.45), and a 1-g increase in mother's intake was associated with a 0.34-g increase in children's intake (95% CI: 0.26–0.41). Finally, Model 3, which further adjusted for the municipality city difference, showed that a 1-g increase in neighborhood women's intake was associated with a 0.20-g increase in children's intake (95% CI: −0.02–0.42), while the coefficient for mother's intake remained the same.

**Table 4:** Association between children's snack intake and the environmental variable.

	Model 1		Model 2		Model 3	
	(n = 748)		(n = 736)		(n = 736)	
	Coefficient	95% CI	Coefficient	95% CI	Coefficient	95% CI
Environmental variable (mean snack intake of neighborhood women excluding children's mothers)	0.41	[0.19, 0.64]	0.23	[0.00, 0.45]	0.20	[−0.02, 0.42]
Mother's snack intake			0.34	[0.26, 0.41]	0.34	[0.27, 0.41]
Sex						
Boys			Ref.		Ref.	
Girls			2.73	[−0.50, 5.96]	2.66	[−0.53, 5.86]
Age			−0.25	[−1.20, 0.71]	−0.31	[−1.26, 0.64]
Existence of siblings below elementary school age						
No or unknown			Ref.		Ref.	
Yes			3.74	[0.22, 7.26]	3.80	[0.31, 7.29]
Mother's educational attainment						
Low			Ref.		Ref.	
Medium			−3.07	[−6.95, 0.81]	−1.41	[−5.33, 2.50]
High			−6.23	[−10.70, −1.75]	−4.40	[−8.91, 0.12]
Missing			−0.75	[−8.96, 7.45]	0.37	[−7.77, 8.51]
Mother's employment status						
Full-time job or self-employed			Ref.		Ref.	
Part-time job or other job			3.76	[−0.86, 8.37]	4.25	[−0.32, 8.82]
Housewife or unemployed			−1.67	[−6.41, 3.07]	−0.42	[−5.15, 4.31]
City						
City 1					Ref.	
City 2					6.21	[1.43, 10.99]
City 3					−1.81	[−6.57, 2.95]
City 4					−3.76	[−7.90, 0.38]
Intercept	24.19	[14.72, 33.66]	19.37	[5.02, 33.72]	19.25	[4.56, 33.94]

CI, confidence interval; Ref, reference category.

## 4 Discussion

To the best of our knowledge, this is the first study to show a cross-sectional association between children's obesogenic health behaviors and the collective behavioral tendencies of their adult neighbors. This study is further characterized by two unique methodological aspects: 1) the use of the behavioral similarity of women's snack intake as a basis for the identification of areal units and 2) the use of spatial regionalization to empirically obtain regional segments as an alternative to predetermined administrative boundaries.

Our main finding was that the collective behavioral tendencies of neighborhood adults as a surrogate index of environmental effects was associated with children's snack intake behavior, even after adjusting for the influence of children's own mothers. This finding indicates that the influence of the out-of-home environment may not be negligible compared with maternal influence. This suggests several theoretical interpretations for the mechanism by which environmental factors affect individual behaviors.

First, the shared physical and social environments in the obtained regional segments may affect the snack intake behaviors of both neighborhood women and children (Flowerdew et al., 2008) as confounders. Because physical and social environmental factors mutually interact, and environment, individual intention, and behavior are reciprocally interrelated in complex ways, it may not be possible to disentangle these mechanisms by simply including built environment information in the analytic model (Diez Roux & Mair, 2010).

An alternative explanation is that the health behaviors of surrounding neighborhood adults may directly influence the health behaviors of elementary school-aged children. Children's health behaviors are socially influenced by familiar people, such as parents and peers, through mechanisms such as observation and social norms (Bevelander et al., 2020; Draper et al., 2015; Rice & Klein, 2019; Salvy & Bowker, 2014). Previous educational and psychological studies have also indicated that non-parental adults in the community can directly or indirectly affect children's behavioral development (Pittman et al., 2020; Scales et al., 2001; The Aspen Institute, 2018, 2019; Whiten et al., 2016). However, few studies have demonstrated similarity in the same behavior between neighborhood adults and schoolchildren (Farrelly et al., 2014; Patchin et al., 2006). Our finding provides empirical evidence for the mechanism of vicarious observation for children's health behaviors, in that snack intake behavior of non-related neighborhood adults may affect the corresponding behavior in children.

The present findings have some methodological and practical implications regarding the use of the regionalization method for social surveys. Our regionalization method produced a higher ICC for women's snack intake than the administrative unit method. This suggests that our approach may capture more “plausible segments” of residents' eating behavioral patterns, which are similar to the “natural areas” empirically identified using a spatially constrained clustering method (Steenbeek & Kreis, 2015). Although traditional multilevel models have conveniently treated predetermined administrative areas as “neighborhood units,” there is no clear reason to assume that neighborhood environmental characteristics that affect people's behaviors differ according to administrative boundaries (Chambers et al., 2017; Takagi, 2013).

Our findings have several policy implications. They suggest that interventions to change children's dietary behaviors could also leverage neighborhood adults rather than focusing more narrowly on children or their families (Perdew et al., 2021; Renzaho et al., 2014; Scaglioni et al., 2011). Recent population-based interventions have adopted such a wider focus to promote children's well-being by targeting both local people and the environment (Organisation for Economic Co-operation and Development, 2019; Kothandan, 2014; World Health Organization, 2012).

Another implication of our findings is that spatial regionalization may help public health practitioners to effectively target neighborhood segments for community interventions. Previous studies have shown that use of appropriate spatial regionalization can capture hidden regional patterns that are sometimes overlooked when using predetermined units (Guo & Wang, 2011; Mu et al., 2014; Wang, 2020; Wang et al., 2012).

### 4.1 Limitations

This study had several limitations. First, it was a cross-sectional study, and thus we could not determine the causal effect of the neighborhood women's behavior on the children's behavior. Longitudinal studies are needed to investigate the causality of this association. Second, our sampling was limited to urban settings, and therefore the generalizability of the findings requires confirmation by studies in other settings. Third, our sample was spatially sparse in each city. Given the possibility that we overlooked some regional segments owing to a lack of spatial data, it is likely that the environmental variable effect was underestimated rather than overestimated. Fourth, we did not try other regionalization methods, and the use of regionalization has room for improvement. (Guo & Wang, 2011; Wang, 2020). Fifth, we had no information about the influence of peers on children, which may have confounded the identified environmental effect (Salvy et al., 2011; Sawka et al., 2015; van Ansem et al., 2015). Sixth, there was a possibility of recall bias owing to the use of a self-administered questionnaire. However, this bias tends to attenuate the observed associations (Willette, 2013). Finally, we did not include a suitable direct measure of observational learning processes or perceived behavioral norms of children. Further studies are needed to isolate the effects of observational learning and perceived norms from the shared environmental effect.

## 5 Conclusions

In this study, we demonstrated an association between children's obesogenic dietary behaviors and the collective behavioral tendencies of their adult neighbors, after adjusting for the mother's behavior. This finding suggests that children's dietary behaviors are affected by their physical and social environments. Our results also indicated that the influence of the out-of-home environment on children's dietary behaviors may not be insubstantial compared with maternal influence, suggesting that changes in the out-of-home environment could modify children's dietary behaviors.

## CRediT authorship contribution statement

**Emiko Yamamoto:** Writing – original draft, Visualization, Validation, Methodology, Formal analysis, Conceptualization. **Daisuke Takagi:** Writing – review & editing, Validation, Methodology, Data curation, Conceptualization. **Hideki Hashimoto:** Writing – review & editing, Validation, Supervision, Resources, Project administration, Methodology, Investigation, Funding acquisition, Data curation, Conceptualization.

## Data statement

The datasets used and/or analyzed during the current study are available from the corresponding author on reasonable request.

## Ethical statement

The study protocol for the J-SHINE project was approved by the Ethics Committee of the Graduate School of Medicine of The University of Tokyo. Written informed consent was obtained from all the participants.

## Funding

This study used data from the Japanese Study on Stratification, Health, Income, and Neighborhood (J-SHINE) project, which was supported by a Grant-in-Aid for Scientific Research on Innovative Areas (No. 21119002) from the Ministry of Education, Culture, Sports, Science, and Technology, Japan. The funding body had no role in the design of the study and collection, analysis, and interpretation of data and in writing the manuscript.

## Declaration of competing interest

The authors have nothing to declare.

## Data Availability

Data will be made available on request.
