# Supplementary material for: Association between snack intake behaviors of children and neighboring women: A population-based cross-sectional analysis with spatial regionalization
Source: SSM Popul Health. 2024 Oct 13;28:101720. doi: 10.1016/j.ssmph.2024.101720 (PMC11539136; doi:10.1016/j.ssmph.2024.101720)
Supplement: Multimedia component 1 [file mmc1.docx]

**Appendix 1:** Technical details for obtaining new regional segments

We obtained geographical information data for municipalities and administrative city blocks from the 2010 Japanese national census data ("Ministry of Land, Infrastructure, Transport and Tourism’s National Land Numerical Information download service," 2010). We matched the obtained geographical information with the city blocks sampled in our survey, and calculated the mean snack intake of the sampled women by each city block as the regional characteristics. We excluded one block because it contained only one woman participant. We relied on the rook contiguity method to define whether the blocks were contiguous or not (i.e., we defined spatial neighbors as sharing a border and not just a vertex of the regions) (Ward and Gleditsch, 2019).

In the JSHINE survey, for each municipality, 60 areas basically according to city blocks were sampled in proportion to the registered population. (in the 2010 census data, city 1 was comprised of 160 city blocks in total; city 2, 269 blocks; city 3, 62 blocks; and city 4, 287 blocks). Because we had both unsampled and sampled blocks in each municipality, we treated the nearest block using the center distance as a contiguous block if a block had no contiguous neighborhood blocks. We set the minimum population of each agglomerate cluster to obtain the peak of the Calinski–Harabasz pseudo F statistic (Calinski and Harabasz, 1974). For city 3, in which the peak of the pseudo F statistic was not obtainable, we set the number of regional segments to 30, as half the number of survey sample units in each municipality. Otherwise, we followed the SKATER procedure for partitioning the regions.

**References**

Calinski, T., Harabasz, J., 1974. A dendrite method for cluster analysis. Communications in Statistics. 3, 1-27. https://doi.org/10.1080/03610927408827101

Ministry of Land, Infrastructure, Transport and Tourism. 2010. Ministry of Land, Infrastructure, Transport and Tourism’s National Land Numerical Information download service. https://nlftp.mlit.go.jp/ksj/index.html (accessed 3 December 2023)

Ward, M.D., Gleditsch, K.S., 2019. Spatial Regression Models Second Edition. SAGE Publications, Inc, United States of America.
