# Supplementary material for: Association between snack intake behaviors of children and neighboring women: A population-based cross-sectional analysis with spatial regionalization
Source: SSM Popul Health. 2024 Oct 13;28:101720. doi: 10.1016/j.ssmph.2024.101720 (PMC11539136; doi:10.1016/j.ssmph.2024.101720)
Supplement: Multimedia component 2 [file mmc2.doc]

**Table Appendix 1.** Background characteristics of the neighborhood female adults whose data were used for the spatial segmentation.

|  |  |  |  |  | **Snack intake (g/1000 kcal)** | | |
| --- | --- | --- | --- | --- | --- | --- | --- |
|  |  |  | Number | % | Mean | Standard deviation | p-value |
| **Total** |  |  | 2275 | 100 | 40.6 | 23.8 |  |
| **Municipality** | |  |  |  |  |  |  |
|  | City 1 |  | 642 | 28.2 | 41.6 | 23.7 | 0.202 |
|  | City 2 |  | 441 | 19.4 | 40.6 | 25.1 |  |
|  | City 3 |  | 508 | 22.3 | 38.7 | 22.9 |  |
|  | City 4 |  | 684 | 30.1 | 41.1 | 23.8 |  |
| **Age (years)** | |  |  |  |  |  |  |
|  | 20–29 |  | 155 | 6.8 | 47.2 | 25.9 | 0.000 |
|  | 30–39 |  | 876 | 38.5 | 40.8 | 24.3 |  |
|  | 40–49 |  | 1038 | 45.6 | 38.9 | 22.9 |  |
|  | ≥50 |  | 206 | 9.1 | 43.4 | 23.6 |  |
| **Educational attainment** | | |  |  |  |  |  |
|  | Low |  | 557 | 24.5 | 38.8 | 24.1 | 0.071 |
|  | Medium |  | 928 | 40.8 | 40.8 | 24.2 |  |
|  | High |  | 704 | 30.9 | 42.1 | 23.3 |  |
|  | Missing |  | 86 | 3.8 | 38.2 | 21.8 |  |
| **Employment status** | | |  |  |  |  |  |
|  | Full-time job or self-employed | | 604 | 26.5 | 40.6 | 23.9 | 0.999 |
|  | Part-time job or other job | | 922 | 40.5 | 40.6 | 24.0 |  |
|  | Housewife or unemployed | | 707 | 31.1 | 40.5 | 23.7 |  |
|  | Missing |  | 42 | 1.8 |  |  |  |
